# Whole Exome Sequencing Reveals Genetic Predisposition in a Large Family with Retinitis Pigmentosa

**DOI:** 10.1155/2014/302487

**Published:** 2014-06-30

**Authors:** Juan Wu, Lijia Chen, Oi Sin Tam, Xiu-Feng Huang, Chi-Pui Pang, Zi-Bing Jin

**Affiliations:** ^1^Division of Ophthalmic Genetics, The Eye Hospital of Wenzhou Medical College, The State Key Laboratory Cultivation Base, No. 270, West Xueyuan Road, Wenzhou 325027, China; ^2^Department of Ophthalmology and Visual Sciences, The Chinese University of Hong Kong, Hong Kong Eye Hospital, 147K Argyle Street, Kowloon, Hong Kong

## Abstract

Next-generation sequencing has become more widely used to reveal genetic defect in monogenic disorders. Retinitis pigmentosa (RP), the leading cause of hereditary blindness worldwide, has been attributed to more than 67 disease-causing genes. Due to the extreme genetic heterogeneity, using general molecular screening alone is inadequate for identifying genetic predispositions in susceptible individuals. In order to identify underlying mutation rapidly, we utilized next-generation sequencing in a four-generation Chinese family with RP. Two affected patients and an unaffected sibling were subjected to whole exome sequencing. Through bioinformatics analysis and direct sequencing confirmation, we identified p.R135W transition in the rhodopsin gene. The mutation was subsequently confirmed to cosegregate with the disease in the family. In this study, our results suggest that whole exome sequencing is a robust method in diagnosing familial hereditary disease.

## 1. Introduction

Retinitis pigmentosa (RP) is an inherited retinopathy with extreme clinical and genetic heterogeneity. Epidemiological study has indicated the prevalence of RP in China to be 1 in 3800 [1]. The common clinical manifestations usually start off as night blindness from adolescence followed by impaired visual fields and visual acuity. Eventually, RP patients suffer from tunnel vision and complete blindness in late stage of the disease. Bone spicule deposits and attenuated retinal vessels are usually observed in the fundus of RP patients. The severity and age of onset of this disease vary dramatically due to diverse genetic contributions [2]. Retinitis pigmentosa can be caused by various defects in many different genes and pathogenicity mechanisms; therefore, it is almost impossible to precisely diagnose this disease with clinical findings alone. RP can be inherited in all inheritance patterns, mainly including autosomal recessive, autosomal dominant, and X-linked recessive patterns [3]. Currently, mutations in 20 genes have been identified for adRP (RetNet). Due to the complex phenotype and genetic heterogeneity of RP, the process of precise molecular diagnosis is still quite difficult and time consuming.

Since the 1980s, tremendous technological advances have been made in identifying genetic mutations contributive to inherited retinal diseases. The continuous development of new techniques not only accelerates the process of identifying pathogenic genes of human genetic diseases, but also provides insights into the mechanisms involved in retinal pathologies [4]. However, most of these techniques are relatively inefficient, expensive, and labor intensive and most importantly do not improve diagnostic efficiency. Protein-coding genes constitute only approximately 1% of the human genome, but they account for 85% of the mutations linked to most genetic disorders [5]. Next-generation sequencing is capable of capturing all protein-coding sequences and therefore allowing the simultaneous analysis of multiple genes and the generation of massive amount of sequence data. These features of next-generation sequencing make it an attractive approach for investigation of coding variations [6]. On account of its remarkable characteristic, next-generation sequencing can contribute to more efficient and accurate molecular diagnosis, especially for those diseases that have no evident symptoms.

In this study, we attempted to identify the candidate pathogenic gene responsible for a Chinese family with RP using whole exome sequencing. In addition, we wanted to evaluate the diagnostic efficiency of using this technique and to establish a correlation between candidate genes and clinical phenotypes.

## 2. Methods

### 2.1. Patient Recruitment

This study complied with the Declaration of Helsinki and was approved by the Ethics Committee of The Eye Hospital of Wenzhou Medical College. Written informed consents were obtained from every patient. We collected information on their detailed family history of RP, such as age of onset and the progress of the disease. Then each participant underwent careful examinations including visual acuity testing using E decimal charts, slit-lamp biomicroscopy, fundus examination, visual field testing, and optical coherence tomography (OCT). The diagnosis of RP was based on the presence of night blindness, typical fundus findings, reduced peripheral visual field, abnormal OCT result, and family history.

We selected two affected participants (III-7 and III-10) and an unaffected sibling (III-4) for whole exome sequencing. Total genomic DNA was extracted from peripheral blood using a DNA Extraction Kit (TIANGEN, Beijing) according to the manufacturer's instructions. DNA was quantified with Nanodrop 2000 (Thermal Fisher Scientific, DE).

### 2.2. Exome Sequencing

For the Illumina HiSeq 2000 platform, Illumina libraries were generated according to the manufacturer's sample preparation protocol. In short, 3 ug of each patient's genomic DNA was fragmented to 100–300 base pairs. According to standard Illumina protocols, we prepared DNA libraries using procedures like end-repair, adenylation, and adapter ligation. DNA fragments were captured by hybridization to the capture panel by using the Exome Enrichment V5 Kit (Agilent Technologies, USA) and sequenced on Illumina HiSeq 2000 Analyzers for 90 cycles per read [7]. The PCR products were purified using SPRI beads (Beckman Coulter) according to manufacturer's protocol. Then the enrichment libraries were sequenced on Illumina Solexa HiSeq 2000 sequencer for paired read of 100–300 bp [8].

### 2.3. Data Filtering and Analysis

After the whole exome sequencing was complete, image analysis, error estimation, and base calling were processed using the Illumina Pipeline to obtain primary data. Firstly, the short paired-end reads were aligned to the reference human genome using SOAPaligner software [9]. Then the mutations in noncoding and intronic regions and the low quality reads were removed from the primary data [10]. SNPs and indels were identified using the SOAPsnp software and the GATK Indel Genotyper [11]. Variants above 1% frequency were also removed and then the remaining variants were analyzed based on their predictive effect of amino acid change and on protein function using PolyPhen, SIFT, PANTHER, and Pmut [12]. Given that this is an inherited disorder in the family, we only kept common variants in the two affected individuals for further analysis.

### 2.4. Confirmation of the Potential Mutations

Once we obtained a list of candidate variants, we amplified the same site of each participant's DNA template and then sequenced the PCR products using Sanger sequencing to confirm the precision of the variants. Then we analyzed Sanger sequencing results by Mutation Surveyor (Softgenetics, PA) and estimated pathogenic effects of the mutations on protein function by Mutation Taster (http://www.mutationtaster.org).

## 3. Results

### 3.1. Phenotypic Determination

**Figure 1:**
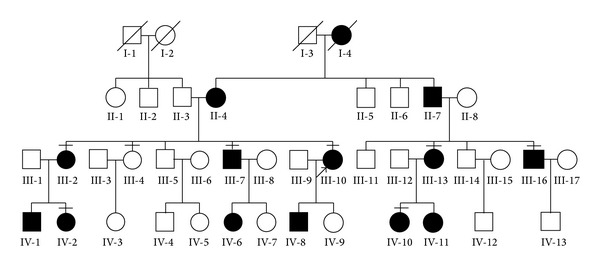
Pedigree of the Chinese family in this study. Filled symbols represent affected patients and unfilled symbols indicate unaffected subjects. The bars over the symbol indicate subjects enrolled in this study.

**Figure 2:**
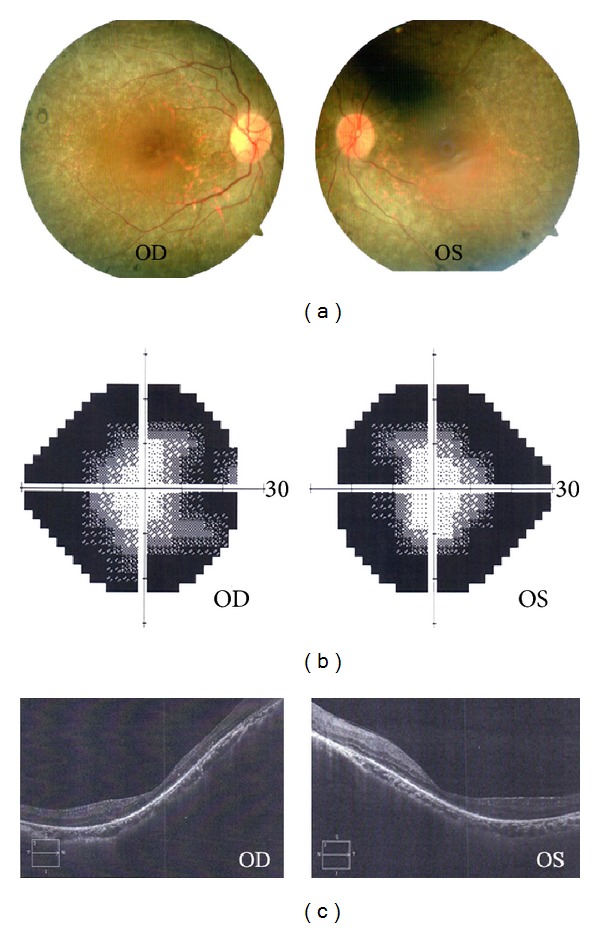
Representative clinical characteristics of the proband. (a) Fundus photographs show bone spicule-like pigmentation and retinal vascular attenuation. (b) Visual field test point locations show the loss of peripheral visual field. (c) Optical coherence tomographic (OCT) images show severe thinning of the photoreceptor inner/outer segment.

The four-generation Chinese family we recruited has 42 members including 14 affected individuals (Figure 1). The inheritance pattern of RP in this family was autosomal dominant. All the affected patients in this study began suffering from severe night blindness before the age of ten. Although they were diagnosed in their youth, most patients started to exhibit characteristic clinical symptoms after the age of thirty and developed total blindness in later life. As they reached the age of onset, their visual acuity reduced quite rapidly and their BCVA dropped to less than 0.3 in their worse eyes. Fundus examinations presented attenuation of the retinal vessels, bone spicule-like pigmentation in the inferior periphery, and retinal pigment epithelium (RPE) atrophy. OCT results clearly displayed severely thin and disorganized inner and outer segment of photoreceptors. Humphrey visual field testing evidently showed serious impairment of peripheral visual field (Figure 2). The unaffected sibling (III-4) has normal vision activity without pathological examination results. Detailed clinical information of the family was summarized in Table 1.

### 3.2. Whole Exome Sequencing Identified the Candidate Gene

The exomes of two affected individuals (III-7 and III-10) in the family were captured and sequenced. Millions of sequencing reads were generated from the two samples. Most of them were aligned to the human reference genome or mutations in noncoding and intronic regions. Mean read depth of target regions was 41.9 X, 44.6 X, and 34.0 X, respectively (see Table S1 in Supplementary Material available online at http://dx.doi.org/10.1155/2014/302487). The remaining variants were then further evaluated by SOAPsnp and their impact on protein function was predicted. At last only one variant in* RHO* (p.R135W) was found among the RP related genes. This mutation and its effect on protein function were previously identified in RP patients by other researchers. We therefore supposed this variant as a candidate mutation responsible for RP.

### 3.3. Sanger Sequencing Confirmation

**Figure 3:**
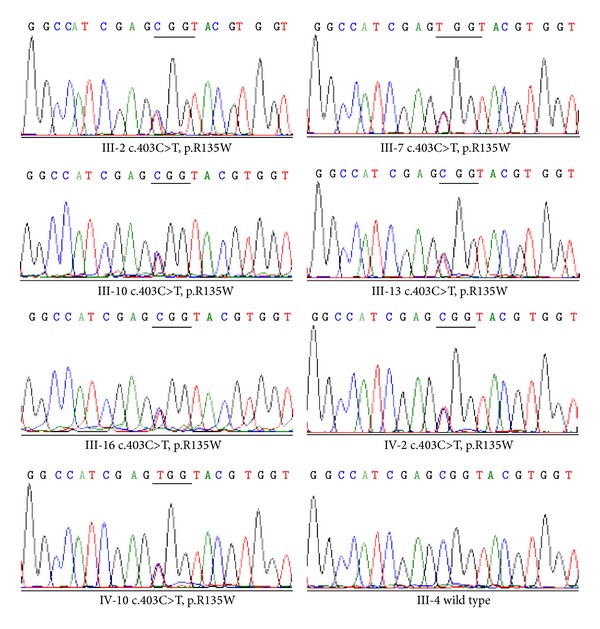
Chromatography of the identified mutation in each patient. Sanger sequencing results obtained from seven affected members (III-2, III-7, III-10, III-13, III-16, IV-2, and IV-10) and an unaffected member (III-4) in the Chinese family.

Candidate variant identified from whole exome sequencing was confirmed using conventional Sanger sequencing to exclude the possibility of false positive. We extracted DNA from the three participants (III-4, III-7, and III-10) and amplified the target fragments. We also amplified and sequenced DNA from five other individuals (III-2, III-13, III-16, IV-2, and IV-10) in the pedigree. Sequence analysis was performed with Mutation Surveyor (Softgenetics, PA). With the exception of the unaffected participant, all of the examined RP patients were confirmed to have the same mutation in their* RHO* gene. We are therefore confident that the mutation (p.R135W) is the disease-causing gene in this family (Figure 3).

### 3.4. Effect of the Mutation

The* RHO *gene, the first gene known to cause RP, encodes the protein rhodopsin, which plays an important role in capturing light and initiating the signal transduction cascade [13]. Mutations in* RHO *usually not only cause dominant RP but also are found in a small fraction of recessive RP [14]. The p.R135W mutation locates in a specific region of the* RHO* gene that impacts the putative second transmembrane segment of the protein, which leads to impaired function of rod photoreceptors [15]. The severity of the disease seems to correlate with the disease stage of the patient and also the molecular basis of the disease. We believe that the p.R135W mutation causes a mild phenotype of night blindness in young age that worsens by middle age.

### 3.5. Mutation Spectrum of the* RHO* Gene

**Figure 4:**
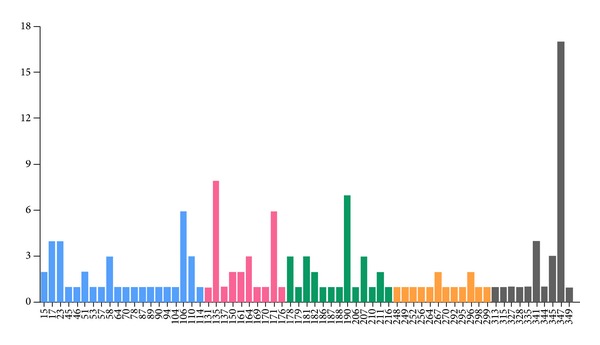
The mutation spectrum of* RHO* gene. The* x*-axis indicates the amino acid position and* y*-axis represents the number of reported mutations. Each color represents an exon: blue: exon 1; pink: exon 2; green: exon 3; orange: exon 4; grey: exon 5.

The mutation distribution in previously reported* RHO* mutations was summarized (Figure 4). Mutations in the* RHO* gene spread out over the entire length of the gene. According to our statistics, amino acid positions 135, 190, and 347 were the top three hot spots in the worldwide RP population.

## 4. Discussion

Due to the genetic and clinical heterogeneity of the inherited retinal diseases, efficient and accurate molecular diagnosis has proven to be quite difficult. Although typical clinical symptoms and information on family history can help promote the diagnosis process, we have yet to narrow down the disease-causing gene from more than 100 candidate genes linked to RP (RetNet).

Current technological advances have allowed once unaffordable techniques like whole exome sequencing to be used as a routine diagnostic tool [16–18]. This sequencing technique covers all of the coding sequences in the human genome and makes it possible to screen a number of genes simultaneously. One of the aims of this study is to evaluate the efficiency of using high-throughput sequencing technique in diagnosing monogenic disorders. We selected two affected patients and one healthy sibling to be subjected to the sequencing. Combining the sequencing data and Sanger sequencing results, we efficiently mapped the candidate mutation to the* RHO* gene at the position 403 on cDNA (c.403C>T), which converts arginine to tryptophan.* RHO* was the first reported gene associated with adRP and its incidence in Chinese individuals was estimated to be relatively high. In our study, the inheritance pattern of the four-generation family follows that of adRP and the clinical symptoms of the affected members are similar to each other. Hence, we propose possible correlations between clinical features of RP and the candidate mutation in* RHO*. Most of the patients in this family suffered night blindness at young age and their visual acuity and vision field worsen from middle age. We speculate that the mutation (p.R135W) may be responsible for these clinical symptoms common within this family.

In conclusion, we have successfully performed whole exome sequencing for screening mutations within a RP family and showed that this technology is an inexpensive and efficient tool for identifying disease-causing mutations. Using whole exome sequencing as part of the routine clinical examination not only will contribute to rapid diagnosis, but also might allow for the discovery of other underlying disease-causing mutations. Furthermore, identifying the disease-causing mutations also opens up the option of using gene therapy to treat the disease.

## Supplementary Material

Shown the quality data of the whole-exome sequencing of three individuals in this study.

## Figures and Tables

**Table 1:** The clinical features of patients in this study.

Subject	Age	Sex	BCVA (OD/OS)	Onset age of night blindness
III-2	50	F	HM/0.1	<10
III-7	40	M	0.05/0.15	<10
III-10	36	F	0.3/0.2	<10
III-13	46	F	0.3/0.3	<10
III-16	42	M	0.3/0.4	<10
IV-2	25	F	0.5/0.5	<10
IV-10	23	F	0.5/0.6	<10

M: male; F: female; BCVA: best corrected visual acuity; OD: right eye; OS: left eye.
