# Supplementary material for: Whole Exome Sequencing Reveals Genetic Predisposition in a Large Family with Retinitis Pigmentosa
Source: Biomed Res Int. 2014 Jun 30;2014:302487. doi: 10.1155/2014/302487 (PMC4102027; doi:10.1155/2014/302487)
Supplement: Supplementary file 1 — Shown the quality data of the whole-exome sequencing of three individuals in this study. [file 302487.f1.doc]

**Supplemental table 1. Quality of the exome resequencing.**

| *Sample* | *III-4* | *III-7* | *III-10* |
| --- | --- | --- | --- |
| Total reads | 60,535,824 | 66,280,246 | 54,852,526 |
| Total yield (bp) | 6,114,118,224 | 6,694,304,846 | 5,540,105,126 |
| Read length (bp) | 101.0 | 101.0 | 101.0 |
| Target regions (bp) | 62,085,286 | 62,085,286 | 62,085,286 |
| Coverage of target regions (more than 1X) | 94.7% | 95.2% | 95.0% |
| Coverage of target regions (more than 10X) | 86.2% | 86.5% | 86.3% |
| Median read depth of target regions | 40.0X | 42.0X | 34.0X |
| Mean read depth of target regions | 41.9X | 44.6X | 34.0X |

Shown the quality data of the whole-exome sequencing of three individuals in this study.
